# The association between atorvastatin administration and plasma total homocysteine levels in renal transplant recipients

**DOI:** 10.15171/jnp.2016.18

**Published:** 2016-04-07

**Authors:** Ali Monfared, Seyyede Zeinab Azimi, Ehsan Kazemnezhad

**Affiliations:** Urology Research Center, Guilan University of Medical Sciences, Guilan, Iran

**Keywords:** Homocysteine, Atorvastatin, Transplantation, Renal, Transplant recipients

## Abstract

**Background:**

Statins improve prognosis in patients with coronary heart diseases by decreasing the incidence of vascular events. Excess prevalence of hyperhomocysteinemia, an independent risk factor of cardiovascular diseases, has been observed in stable renal transplant recipients (RTRs).

**Objectives:**

The objective of our study was to evaluate the association between atorvastatin administration and plasma total homocysteine (tHcy) levels in RTRs.

**Patients and Methods:**

We performed a retrospective cross-sectional study in 148 cyclosporine A (CsA) treated stable RTRs. We compared tHcy level and other demographic and clinical variables in RTRs with and without atorvastatin.

**Results:**

58.1% of the 148 RTRs were treated with atorvastatin (20-40 mg/day). Mean tHcy levels were lower in patients treated with atorvastatin compared to nonusers (14.80 ± 5.13 µmol/l versus 16.95 ± 7.87 µmol/l, *P* = 0.04). The comparison of 85 patients treated with atorvastatin and 61 non-users revealed that those subjects with atorvastatin were older, with higher estimated creatinine clearance and elevated body mass index (BMI). They were more likely to have higher systolic blood pressure and CsA trough level (C0). The association between lower tHcy levels and atorvastatin use was confirmed in the multivariate regression model (*P* = 0.004). However tHcy levels were independently and negatively associated with serum folate (*P* = 0.0001) and vitamin B12 levels (*P* = 0.001) and positively with serum BUN (*P* = 0.001) and diastolic blood pressure (*P* = 0.024) as well.

**Conclusions:**

These data support the association between lower tHcy levels and atorvastatin administration in RTRs. Further clinical trials are recommended to clarify homocysteine lowering effect of atorvastatin.

**Implication for health policy/practice/research/medical education:**

Cardiovascular disease is one of the most common causes of mortality in dialysis patients. Also, hyperhomocysteinemia isconsidered as a risk factor for atherosclerosis that in dialysis patients is more prevalent comparing with other patients. Hence,risk of atherosclerosis and cardiovascular death would be reduced in case of hyperhomocysteine amendment.

## 1. Background

Cardiovascular disease is the main cause of death in renal transplant recipients (RTRs) with a functioning allograft (1,2). Among the contributing factors of the increased risk of ischemic heart disease and death from cardiovascular diseases after transplantation, older age of the recipients, obesity, hypertension, hyperlipidemia, diabetes mellitus, and smoking appear to be more predominant (3).

Studies show that stable RTRs have an excess prevalence of hyperhomocysteinemia (4-6). Moreover homocysteine is supposed to contribute to oxidative stress and endothelial damage (7). Deficiency of some water soluble vitamins, especially vitamin B6, B12, and folic acid may result in hyperhomocysteinemia (8).

Statins (3-hydroxy-3-methylglutaryl coenzyme A reductase inhibitors [HMG-CoA reductase]) improve coronary heart diseases prognosis in patients by decreasing the incidence of vascular events. They have favorable pleiotropic effects, including antithrombotic, protecting endothelial functions, changing thrombus formation, altering platelet aggregation and enhancing fibrinolysis (9,10). In addition, atorvastatin is increasingly used in solid organ transplant recipients treated with cyclosporine A (CsA) (11).

However, the effects of statins on homocysteine, are not yet well established (12) and the mechanisms of the antithrombotic action of statins are unclear (9). Controversy exists about the effects of statins on homocysteine levels in renal transplant patients (8).

## 2. Objectives

The purpose of this study was to determine the association between atorvastatin administration and plasma total homocysteine (tHcy) levels in stable RTRs and to evaluate other associated factors.

## 3. Patients and Methods

### 3. 1. Study population

We retrospectively analyzed recorded databases from 148 stable RTRs in a transplant center in north of Iran. According to their records, immunosuppressive regimen of patients consisted of cyclosporine (CsA; Iminoral, Zahravi, Iran) 2.5-5 mg/kg/day divided in a twice daily dosage, prednisolone 5-10 mg/day orally and mycophenolate mofetil (Cellcept, Roshe, Basel, Switzerland) 1000 mg twice daily.

All the atorvastatin (Sobhan, Rasht, Iran) consumers took it at least since their transplantation time. For the majority of our patients, getting the exact time of prescribing the drug was inaccessible. The prescribed dosing of atorvastatin was on the basis of our physicians’ discretion.

All the patients received grafts from living donors. All RTRs were over 18 years old, with first renal transplantation and post-transplant time of at least 6 months. Neither of them had liver diseases, psoriasis, rheumatoid arthritis, any kind of cancer nor taking B vitamins or methotrexate.

Cockcroft-Gault formula was conducted to estimate the endogenous creatinine clearance (eCrCl).

Blood pressure was measured in a standardized manner using a calibrated mercury sphygmomanometer, with the patient sitting for at least 5 minutes prior to measurement. Body mass index (BMI) was also measured for all participants.

The etiology of end-stage renal disease consisted of chronic glomerulonephritis (n=45, 30.8%), diabetic nephropathy (n=12, 8.2%), nephrosclerosis (n=15, 10.3%), obstructive nephropathy (n=19, 13%), chronic pyelonephritis (n=1, 0.7%), polycystic kidney disease (n=7, 4.8%), tubulointerstitial nephritis (n=6, 4.1%), analgesic nephropathy (n=3, 2.1%), focal segmental glomerular sclerosis (n=2, 1.4%) and Alport syndrome (n=1, 0.7%).

### 3.2. Laboratory assessments

The laboratory tests were done between April 2011 and January 2012. Blood samples were drawn from the antecubital vein after an overnight (10-14 hours) fast. A fasting blood sample collected into an EDTA-anticoagulated tube for measuring plasma homocysteine for each patient. The blood sample was centrifuged immediately after collection to separate the plasma. Then the separated plasma was deeply frozen (-20^o^C). Total plasma homocysteine was assessed by high performance liquid chromatography (HPLC) with fluorescence detection. Whole blood CsA, serum vitamin B12 and folate concentrations were measured by radioimmunoassay. Whole blood CsA was measured twice, first for assessing CsA trough level in fasting blood (C0) and next, two hours after administration of CsA (C2). All routine biochemistry was performed using colorimetric methods. LDL cholesterol levels were calculated by Friedewald formula: LDL cholesterol = total cholesterol – (HDL cholesterol + triglycerides/5) (9).

All measurements were performed in a single laboratory. Hyperhomocysteinemia (hyperHcy) was defined as plasma tHcy level greater than 12 μmol/l. The normal values for vitamin B12 and folate were 120-970 ρg/ml and 3.1-17.5 ng/ml respectively (13).

### 3.3. Ethical issues

1) The research followed the tenets of the Declaration of Helsinki. 2) This project was approved by ethics committee of Guilan University of Medical Sciences, Iran.

### 3.4. Statistical analysis

Continues variables were expressed as mean values with Standard deviations (SDs). In the univariate analysis, we applied χ^2^ test for categorical data, student’s *t* test and Pearson’s correlation coefficient for the quantitative variables. In the multivariate analysis, multiple linear regression models by stepwise method (Entry = 0.05, Removal = 0.1) for determining association between atorvastatin and tHcy levels was applied. Statistical significance was defined as *P* value less than 0.05. The SPSS version 18 was used for analysis of data.

## 4. Results

The demographic and laboratory parameters of the studied patients are shown in Table 1. Among 148 of participants (87 male and 61 female, with mean age of 44.07±11.52 years), 86 of them (58.1%) used atorvastatin with a mean dose of 20-40 mg. Forty-nine of patients with atorvastatin (57%) were male and 37 were female (43%). The mean age of RTRs with atorvastatin was 46.78±10.56 years.

**Table 1:** Demographic and clinical characteristics of renal transplant recipients

**Subject characteristics**	**Mean ± SD**
Age (years)	44.07 ± 11.52
Gender (M/F)	87/61
Smoking (%)	2.7%
Post-transplant diabetes mellitus (%)	18.2%
Dialysis duration (months)	12.5 ± 12.87
Transplant duration (months)	53.97 ± 40.48
BMI (kg/m^2^)	26.96 ± 4.12
SBP (mm Hg)	128.38 ± 16.95
DBP (mm Hg)	78.85 ± 12.41
tHcy (μmol/L)	16.25 ± 8.32
Folate (ng/mL)	13.71 ± 4.82
Vitamin B12 (pg/mL)	379.99 ± 185.79
CsA dose (mg/day)	168.92 ± 49.11
CsA trough level (C0; μmol/L)	159.12 ± 84.33
PostdoseCsA level (C2; μmol/L)	604.69 ± 260.85
BUN (mg/dl)	22.88 ± 10.37
Creatinine (mg/dl)	1.34 ± 0.57
Uric acid (mg/dl)	5.76 ± 1.44
Albumin (gr/dl)	4.56 ± 0.57
FBS (mg/dl)	95.21 ± 21.28
Total cholesterol (mg/dl)	176.54 ± 41.12
HDL (mg/dl)	45.84 ± 11.03
LDL (mg/dl)	95.20 ± 31.44
TG (mg/dl)	183.06 ± 95.22
CRP ≥ 8 mg/L (%)	7.4%
Estimated creatinine clearance (mL/min)	70.31 ± 18.68
Atorvastatin administration (%)	86 (58.1%)

Abbreviations: SBP, systolic blood pressure; DBP, diastolic blood pressure; BMI, body mass index; CsA, cyclosporine; BUN, blood urea nitrogen; FBS, fasting blood sugar; HDL, high-density lipoprotein; LDL, low-density lipoprotein; TG, triglyceride; CRP, C-reactive protein.

Table 2 shows that among all of the nonimmunosuppresive medications used by patients, the mean of Hcy level differed statistically significant in patients with atorvastatin. In the multivariate analysis using multiple linear regression model by stepwise method (Entry=0.05, Removal=0.1), adjusted for confounders (other nonimmunosuppresive medication) still atorvastatin was correlated with tHcy level (Table 3).

**Table 2:** Comparison of mean ± SD of Hcy level in different drugs usage

**Drug**	**Number**	**HCY level (Mean ± SD)**	**P value**
Calcium-D			0.76
Yes	96	15.82 ± 5.99	
No	50	15.48 ± 7.39	
Rocaltrol			0.10
Yes	12	12.78 ± 3.57	
No	134	15.96 ± 6.63	
Atrovastatin			0.04
Yes	85	14.80 ± 5.13	
No	61	16.95 ± 7.87	
Diltiazem			0.97
Yes	61	15.68 ± 7.09	
No	87	15.72 ± 6.05	
Gemfibrozil			0.45
Yes	10	14.23 ± 4.40	
No	‏136	15.81 ± 6.61	
Losartan			0.28
Yes	27	16.90 ± 4.94	
No	119	15.43 ± 6.77	
Atenolol			0.58
Yes	35	16.22 ± 4.99	
No	‏111	15.54±6.90	
Metoral			0.70
Yes	28	15.74 ± 5.87	
No	117	15.73 ± 6.66	
Amlodipine			0.69
Yes	20	16.24 ± 5.44	
No	‏126	15.62 ± 6.65	

Abbreviation: SD, standard deviation‏.

**Table 3:** Regression coefficient of effect of atorvastatin usage on Hcy level according to multiple linear regression models

**Model**	**B Unstandardized coefficients ± SE**	**β Standardized coefficients**	**t**	**P**	**95% CI for B**
Constant	12.22±2.06		5.93	0.000	8.15 to 16.29
Atorvastatin	2.85±1.37	0.17	2.08	0.040	0.14 to 5.59

Abbreviation: SE, standard error.

Distribution of Hcy which was checked by Kolmogorov-Smirnov test was normal (*P*>0.05, only one patient from the group of atorvastatin users and one from nonusers had out layer tHcy level which were deleted in the analysis).

### 4.1. Univariate analysis

We found strong inverse correlations of tHcy concentration with age (*P*=0.003, r=-0.25), folic level (*P*=0.001, r=-0.35), vitamin B12 (*P*=0.04, r=-0.017) and also with eCrCl (*P*=0.001, r=-0.33). Additionally, significant correlations of tHcy level with BUN (*P*=0.001, r=0.44) and serum creatinine (*P*=0.001, r=0.43) was found.

In univariate analysis, significant positive associations of atorvastatin administration with age (*P*=0.001), systolic blood pressure (*P*=0.009), BMI (*P*=0.001), CsA trough level (C0) (*P*=0.04), estimated creatinine clearance (*P*=0.03) and tHcy (*P*=0.04) was found (Table 4).

**Table 4:** Demographic and clinical data of renal transplant recipients according to atorvastatin administration

	**With atorvastatin** **(n = 85)**	**Without atorvastatin** **(n = 61)**	**P value**
Age (years),mean ± SD	46.06 ± 10.29	40.38 ± 11.91	0.001
Gender (M/F)	49/36	37/24	0.72
Creatinine (mg/dL), mean ± SD	1.28 ± 0.48	1.44 ± 0.68	0.09
BUN (mg/dL), mean ± SD	22.34 ± 9.13	23.72 ± 11.99	0.43
Glomerulonephritis, n (%)	23 (27.1%)	20 (33.9%)	0.45
Smoking, n (%)	2 (2.4%)	2 (3.3%)	0.73
Post-transplant diabetes mellitus, n (%)	21 (24.7%)	6 (9.8%)	0.02
Uric acid (mg/dL), mean ± SD	5.67 ± 1.39	6.96 ± 8.47	0.25
CsA dose (mg/day), mean ± SD	166.47 ± 47.80	172.95 ± 51.70	0.44
SBP (mm Hg), mean ± SD	131.59 ± 17.93	124.18 ± 14.81	0.009
DBP (mm Hg), mean ± SD	79.94 ± 12.97	77.30 ± 11.75	0.21
Dialysis duration (months), mean ± SD	12.19 ± 13.07	12.42 ± 12.58	0.92
Transplant duration (months), mean ± SD	54.25 ± 38.74	52.57 ± 41.85	0.80
BMI (kg/m^2^), mean ± SD	28.04 ± 4.05	25.59 ± 3.81	0.001
Folate (ng/ml), mean ± SD	13.61 ± 4.51	13.96 ± 5.28	0.68
Vitamin B12 (ρg/ml), mean ± SD	366.47 ± 162.34	402.40 ± 215.17	0.27
CsA trough level (C0; μmol/L), mean ± SD	171.23 ± 83.70	142.60 ± 84.20	0.04
Postdose CsA level (C2; μmol/L), mean ± SD	636.10 ± 260.77	565.34 ± 260.60	0.10
Albumin (g/dL), mean ± SD	4.57 ± 0.53	4.55 ± 0.64	0.83
FBS (mg/dL), mean ± SD	97.89 ± 23.99	91.66 ± 16.68	0.08
Total cholesterol (mg/dL), mean ± SD	174.48 ± 37.36	179.87 ± 46.53	0.44
HDL (mg/dL), mean ± SD	47.09 ± 11.23	43.93 ± 10.25	0.08
LDL (mg/dL), mean ± SD	91.18 ± 28.72	101.13 ± 34.63	0.07
TG (mg/dL), mean ± SD	190.76 ± 89.76	173.75 ± 103.10	0.29
CRP > or =8 mg/l, n (%)	1.08 ± .28	1.07 ± 0.25	0.71
Estimated creatinine clearance (mL/min), mean ± SD	73.41 ± 18.46	66.21 ± 18.42	0.02
tHCY (μmol/L), mean ± SD	14.80 ± 5.13	16.95 ± 7.87	0.04

Abbreviations: SBP, systolic blood pressure; DBP, diastolic blood pressure; BMI, body mass index; CsA, cyclosporine; BUN, blood urea nitrogen; FBS, fasting blood sugar; HDL, high-density lipoprotein; LDL, low-density lipoprotein; TG, triglyceride; CRP, C-reactive protein.

RTRs receiving atorvastatin in comparison with those not receiving atorvastatin, were older, with lower levels of tHcy (14.80 ± 5.13 µmol/l versus 16.95 ± 7.87 µmol/l, *P*=0.04). Also BMI was higher in patients who administered atorvastatin (28.04±4.05 kg/m^2^versus 25.59±3.81 kg/m^2^, *P*=0.001). Moreover they demonstrated higher systolic blood pressure and CsA trough level (C0). In addition atorvastatin administration was associated with a higher eCrCl (Table 4). Serum folic acid and vitamin B12 levels were similar in both groups.

### 4.2. Linear regression model

Table 3 shows a multivariate linear regression analysis by stepwise method (Entry=0.05, Removal=0.1) in which we only compared the effects of different drugs used by patients on tHcy level without adjusting for other parameters such as clinical and demographics.

Then we established *P*<0.1 (Entry=0.05, Removal=0.1) for entering all variables (including drugs, demographic and clinical factors) into the complete model of multivariate linear regression model, to determine the effects of more than one variable on a continuous dependent variable (tHcy). It was revealed that the only factors which were independently associated with tHcy serum levels included serum folate, serum vitamin B12, serum blood urea nitrogen (BUN), diastolic blood pressure and atorvastatin administration (Table 5).

**Table 5:** Linear regression model of tHcy levels: adjusted for demographic and clinical factors and drugs usage

**Model**	**B Unstandardized coefficients ± SE**	**β Standardized coefficients**	**t**	**P**	**95% CI for B**
Constant	9.91 ± 4.99		1.98	0.049	0.026 ± 19.788
Serum folic level	-0.61 ± 0.13	-0.35	-4.83	0.000	-0.855 ± -0.358
BUN	0.21 ± 0.06	0.26	3.64	0.000	0.097 ± 0.329
Serum vitamin B12 level	-0.01 ± 0.003	-0.24	-3.34	0.001	-0.018 ± -0.005
Atrovastatin	-3.68 ± 1.25	0.21	-2.95	0.004	-1.209 ± -6.146
DBP	0.11 ± 0.05	0.17	2.29	0.024	0.016 ± 0.215

Abbreviations: DBP, diastolic blood pressure; BUN, blood urea nitrogen‏.

RTRs treated with atorvastatin, had 3.68 folds lower levels of homocysteine in comparison of those not treated with atorvastatin (95% CI: -1.21±-6.15). In addition patients with higher serum folate or vitamin B levels showed lower tHcy level. On the other hand, higher homocysteine levels were associated with higher serum BUN and higher diastolic blood pressure (Table 5).

## 5. Discussion

RTRs experience an increased risk for atherosclerotic cardiovascular disease (1,14). Hyperhomocysteinemia is regarded as an independent risk factor for vascular diseases, and homocysteine is supposed to contribute to oxidative stress and endothelial damage (7).

Statin therapy is an established intervention to diminish the risk of acute events in patients suffering from cardiovascular diseases (7). In addition, statins should be considered the first-line treatment of dyslipidemia observed in renal disease patients (14).

Apart from their lipid-lowering capacity, statins exert anti-inflammatory and antioxidant property as well. As cellular immune activation and oxidative stress play a major role in the pathogenesis of cardiovascular diseases, the anti-inflammatory capacity of statins, to some extent could be responsible for the beneficial effects observed in patients (7,15).

Decreased epithelial progenitor cells and their impaired function were shown to correlate with endothelial dysfunction and atherosclerosis (16). Hcy increases nicotinamide adenine dinucleotide phosphate (NADPH), while atorvastatin may inhibit Hcy induced activation of NADPH oxidase and exert cellular antioxidant effects (17).

Data on early statin use in kidney transplant patients receiving CsA in our region is lacking, and this observational study adds valuable information to existing knowledge.

In our study, atorvastatin administration was significantly associated with lower mean levels of plasma tHcy.

Schroecksnadel et al suggested that statins may prevent homocysteine accumulation in the blood via immunosuppression. They could show the down-regulating effects of atorvastatin on homocysteine formation in vitro (7).

Although, some studies proposed that statins can reduce plasma tHcy levels (7,18), but many failed to demonstrate this effect (8,9,12,19-21).

Luftjohann et al reported a significant decrease in plasma tHcy levels after high doses of simvastatin 80 mg/day for 24 weeks in patients with hypercholesterolemia (18). On the contrary, Miltiadous et al showed that the administration of atorvastatin 40 mg/day for 10 weeks did not affect tHcy levels in 61 patients with hyperlipidemia (12). In addition, Dierkes and colleagues showed that some lipid-lowering and antihypertensive drugs could increase serum levels of homocysteine (22). Also Ozbay et al found that atorvastatin leaded to a mild increase in plasma tHcy levels of patients with mixed hyperlipidemia and it may be explained by its effect on liver function (23).

Shojaei et al showed contradictory results to our findings. Serum homocysteine concentration was 33% higher than normal in the hemodialysis patients who took statin, folic acid, and vitamin B6 (8).

Navarro and colleagues reported that although atorvastatin administration to diabetic patients on hemodialysis was associated with improvement of lipid profile and reduction of high sensitive CRP, but it did not change Hcy levels significantly (20).

Of note, Van der Loo et al showed that treatment with 80 mg atorvastatin resulted in an increase of homocysteine plasma levels in the presence of rather elevated levels of folic acid among patients with peripheral arterial disease (24).

In the present study, we also found that as well as atorvastatin administration, serum folate and vitamin B12 could independently associate with mean tHcy levels in transplant recipients.

MacMahon et al showed that in 141 post-myocardial infarction patients with primary hypercholesterolemia, divided into two groups. The first group took 80 mg/day simvastatin with 2 mg folic acid/0.8 mg vitamin B12 daily and the second group just took 2 mg folic acid/0.8 mg vitamin B12 daily (without simvastatin). In both groups homocysteine level decreased similarly, this reduction in homocysteine was 25.3% and 23.1%, respectively. They also reported no detectable antagonistic effects at the time of administrating simvastatin and folic acid/vitamin B12 concomitantly (25).

Our observational study had a prominent limitation: obtaining the duration of atorvastatin administration was inaccessible precisely, but at least all the RTRs administered statin since their transplantation (with mean transplantation duration of 53.97±40.48 months [range, 6-186 months]).

The Assessment of LEscol Renal Transplantation (ALERT) study is the only randomized controlled trial of statins in renal transplant patients. This trial failed to found any statistically significant advantage of fluvastatin compared to placebo in achieving primary outcomes of reduction in major adverse cardiac events (MACE), the overall mortality or graft survival (26). However, a 2 year extension of the ALERT trial reported that patients randomized to the fluvastatin group had a reduced risk of MACE; still without significant difference in overall mortality and graft loss (27).

The findings of the ALERT trial suggested that the timing of statin use may be determinant for the extent of the non-immune benefits of statins. A greater reduction in major cardiac events had been observed in those patients initiating statin therapy earlier (years 0-2) than those initiating statin therapy later (>6 years) following transplantation (27).

Although cyclosporine causes hypertension and increases cholesterol levels in RTRs (28), Asberg et al indicated that treatment with atorvastatin in CsA treated RTRs is effective in both reducing atherogenic lipids and improving endothelial function through increasing nitric oxide concentration in peripheral plasma (29). They later showed that bilateral pharmacokinetic interaction between atorvastatin and CsA resulted in six fold higher plasma HMG-CoA reductase inhibitory activity after 4 weeks of treatment with atorvastatin 10 mg/day, but systemic exposure of CsA only moderately decreased. Mild cholestasis, associated with CsA therapy, which interfere atorvastatin excretion into the bile or its uptake into hepatocytes, could be the plausible explanations (11).

So the predominant use of cyclosporine (that increases atorvastatin level) at our transplant center, may also partially explain the relatively low tHcy levels in our patient population who took statin.

We also found an association between atorvastatin administration and high BMI in our single center RTRs. A possible explanation could be relatively high prevalence of statin usage in patients with metabolic syndrome regarding their hyperlipidemic state.

In our study, patients taking atorvastatin were generally older and the reasons for initiation of statin therapy were not the same for all the patients. There are two possible explanations for our findings: patients having any indication for statin therapy were older and the prevalence of hyperlipidemia in older age group is higher.

## 6. Conclusions

We observed the association between atorvastatin administration with older age, higher BMI, higher systolic blood pressure, higher eCrCl, lower tHcy levels and higher CsA concentrations. However, after adjusting multiple variables serum folate, vitamin B12, BUN, diastolic blood pressure and atorvastatin administration remained independent associated factors of tHcy levels. These findings would expand the existing knowledge by determining the associations of atorvastatin usage in our RTRs largely managed with a cyclosporine-based immunosuppressive regimen. There is still a need for large, well designed randomized trials in renal transplant patients to establish a positive homocysteine lowering role of statins in this particular population.

## Limitations of the study

Our observational study had major limitations such as lack of the precise duration of atorvastatin administration by patients individually and the design of study which was a retrospective one.

## Acknowledgments

This study is adapted from the M.D. thesis of Seyyede Zeinab Azimi (Thesis number # 1383).

## Authors’ contribution

AM; participated in research design, the writing of the paper, and the performance of the research. SZA; participated in the writing of the paper, the performance of the research, and data analysis. EK; participated in new reagents or analytic tools and data analysis.

## Conflicts of interest

The authors declared no competing interests.

## Funding/Support

The authors declare that they have no competing financial interests in relation to the work described. This research was supported by Urology Research Center, Guilan University of Medical Sciences, Rasht, Iran.
